# Current production by non-methanotrophic bacteria enriched from an anaerobic methane-oxidizing microbial community

**DOI:** 10.1016/j.bioflm.2021.100054

**Published:** 2021-06-15

**Authors:** S. Berger, D.R. Shaw, T. Berben, H.T. Ouboter, M.H. in ’t Zandt, J. Frank, J. Reimann, M.S.M. Jetten, C.U. Welte

**Affiliations:** a Institute for Water and Wetland Research, Department of Microbiology, Radboud University, Nijmegen, the Netherlands; b Biological and Environmental Science and Engineering Division, Water Desalination and Reuse Research Center, King Abdullah University of Science and Technology, Thuwal, Saudi Arabia; c Netherlands Earth System Science Center, Utrecht University, Utrecht, the Netherlands; d Soehngen Institute of Anaerobic Microbiology, Radboud University, Nijmegen, the Netherlands

**Keywords:** Extracellular electron transfer, Microbial community, Acetate, Cytochromes, Zoogloea, ANME-2d, Methanoperedens

## Abstract

In recent years, the externalization of electrons as part of respiratory metabolic processes has been discovered in many different bacteria and some archaea. Microbial extracellular electron transfer (EET) plays an important role in many anoxic natural or engineered ecosystems. In this study, an anaerobic methane-converting microbial community was investigated with regard to its potential to perform EET. At this point, it is not well-known if or how EET confers a competitive advantage to certain species in methane-converting communities. EET was investigated in a two-chamber electrochemical system, sparged with methane and with an applied potential of +400 mV versus standard hydrogen electrode. A biofilm developed on the working electrode and stable low-density current was produced, confirming that EET indeed did occur. The appearance and presence of redox centers at −140 to −160 mV and at −230 mV in the biofilm was confirmed by cyclic voltammetry scans. Metagenomic analysis and fluorescence *in situ* hybridization of the biofilm showed that the anaerobic methanotroph ‘*Candidatus* Methanoperedens BLZ2’ was a significant member of the biofilm community, but its relative abundance did not increase compared to the inoculum. On the contrary, the relative abundance of other members of the microbial community significantly increased (up to 720-fold, 7.2% of mapped reads), placing these microorganisms among the dominant species in the bioanode community. This group included *Zoogloea* sp., *Dechloromonas* sp., two members of the Bacteroidetes phylum, and the spirochete *Leptonema* sp. Genes encoding proteins putatively involved in EET were identified in *Zoogloea* sp., *Dechloromonas* sp. and one member of the Bacteroidetes phylum. We suggest that instead of methane, alternative carbon sources such as acetate were the substrate for EET. Hence, EET in a methane-driven chemolithoautotrophic microbial community seems a complex process in which interactions within the microbial community are driving extracellular electron transfer to the electrode.

## 1 Introduction

In their natural habitats, most microorganisms live in complex communities in which a multitude of interactions with other community members occur. This includes competition for nutrients and may be similarly important to cross-feeding of essential nutrients or removal of toxic intermediates. Especially in anoxic ecosystems syntrophic microbial interactions are needed to break down organic matter and prevent build-up of organic acids and hydrogen [1,2]. The end product of such an anaerobic food web, biogas or methane, can be oxidized to carbon dioxide, dependent on the presence of suitable electron acceptors.

Anaerobic oxidation of methane (AOM) was first described in consortia of anaerobic methanotrophic archaea (ANME) and sulfate-reducing bacteria (SRB) in marine environments [[3], [4], [5], [6], [7]]. In 2006, a new type of ANME belonging to the ANME-2d subgroup was described, and experimental evidence confirmed that these archaea oxidize methane with concomitant nitrate reduction [8]. In the same culture a bacterial anaerobic methanotroph was identified as ‘*Ca.* Methylomirabilis (M.) oxyfera’ [9]. Instead of nitrate, ‘*Ca.* M. oxyfera’ uses nitrite as electron acceptor and converts it to dinitrogen gas. The ANME-2d archaeon ‘*Ca.* Methanoperedens BLZ2’ and ‘*Ca.* M. oxyfera’ were dominant in our enrichment culture. The community included approximately 40 different bacteria that were almost all part of the Alpha-, Beta- and Gammaproteobacteria and the Bacteroidetes phyla. In this enrichment culture, methane is the primary energy source and is converted to CO_2_ by ‘*Ca.* Methanoperedens BLZ2’. Electrons are transferred to nitrate, which results in nitrite production. This provides the substrate for ‘*Ca.* M. oxyfera’, which oxidizes methane and concomitantly reduces nitrite to dinitrogen gas, thereby preventing build-up of toxic concentrations of nitrite. In addition to CO_2_, more reduced carbon compounds might be derived from microbial methane oxidation. It has been shown that ‘*Ca.* Methanoperedens’ can produce acetate, which is excreted into its environment [10]. Furthermore, the pathway used by ‘*Ca.* M. oxyfera’ has been shown to potentially produce methanol [11]. As the other bacterial community members can most likely not directly metabolize methane, they can probably sustain themselves by using acetate, methanol and other compounds, for example those derived from decaying biomass.

We were interested how the microbial community would respond to the presence of an electrode as terminal electron acceptor instead of nitrate as this would circumvent the need for effective nitrite scavengers. The ability to perform extracellular electron transfer (EET) has been described in several bacterial and two archaeal species [12,13]. Furthermore, it has been postulated that EET occurs in ANMEs. Attempts to uncouple ANME and SRB with diffusible substrates such as H_2_ and formate were so far unsuccessful [14]. In 2015, it was proposed that direct electron transfer fitted best with a generalized model of electric conductivity in two different ANME-SRB consortia [15]. Potentially conductive pili were reported to connect the SRB HotSeep-1 to a thermophilic ANME-1 archaeon [14]. The decoupling of ANME-2a and ANME-2c from their bacterial partner was finally achieved by using anthraquinone-2,6-disulfonate (AQDS) as an alternative extracellular electron acceptor [16]. Similarly, for ANME-2d in aquatic and terrestrial environments, AOM is supported by the presence of acceptors that need an EET machinery. These include humic substances or biochar as well as Fe(III), Mn(III) and Mn(IV) [[17], [18], [19], [20]]. Furthermore, ‘*Ca*. Methanoperedens ferrireducens’ was enriched in a bioreactor inoculated with sediment sample and fed with methane and ferrihydrite [21]. A similar enrichment strategy was used to enrich for ‘*Ca.* Methanoperedens manganireducens’ using birnessite instead of ferrihydrite [22]. In a culture enriched in ‘*Ca*. Methanoperedens MPEBLZ’, methane oxidation with the concomitant reduction of nanoparticulate Fe(III) and Mn(IV) was demonstrated [23]. *c*-type cytochromes are widespread electron transfer proteins that have been shown to participate in multiple EET pathways [24]. An unusually high number of *c*-type cytochromes can be found encoded across ANME-2d genomes ranging from 3 to 49 multiheme cytochromes with an average of 26 making this a possible route for EET in these organisms [25]. For the anaerobic bacterial methane oxidizer ‘*Ca*. M. oxyfera’, currently there is no evidence for the capability to perform EET [23]. Also other methanotrophs have been proposed to perform EET. Aerobic methanotrophs have been proposed to be able to reduce minerals under hypoxic conditions [26]. [27] showed EET by the methanotrophic bacterium *Methylococcus capsulatus* (Bath).

Extracellular electron transfer has been demonstrated in many bacterial species. Model organisms that perform direct electron transfer (DET) include the Gammaproteobacterium *Shewanella oneidensis* [28] and Deltaproteobacteria of the genus *Geobacter* [[29], [30], [31]]. Here, electrons are transferred via a series of cytochromes from quinols in the cytoplasmic membrane to the periplasm, across the outer membrane until they are released to an extracellular acceptor via a solvent-exposed heme group. Porin proteins facilitate the passage of electrons across the outer membrane. Another EET mechanism has been described in bacteria, such as *Shewanella* spp. [32], *Klebsiella pneumoniae* [33], *Citrobacter* strain Z7 [34] and *Geothrix fermentans* [35]. Here, soluble mediators such as flavins and quinols can mediate transfer of electrons between cells and an electrode.

In the present study, an anode in a bioelectrochemical system, poised at +400 mV was used as sole electron acceptor for an anaerobic methane-oxidizing microbial community. We were interested in which community members were able to perform EET and if a shift in community composition would occur due to the presence of an electrode as terminal electron acceptor. It was demonstrated that indeed low density current was produced. Analysis of the anode biofilm showed that ‘*Ca.* Methanoperedens BLZ2’ was a significant member of the community. However, its relative abundance did not increase in comparison to the inoculum. In contrast, several bacterial species (*Zoogloea* sp., *Dechloromonas* sp. and one member of the Bacteroidetes) increased in relative abundance and their MAGs encoded proteins potentially involved in EET. Possibly, different carbon sources derived from metabolic cross-feeding or decaying biomass were driving the current production. We suggest that EET was most likely performed by these three bacteria.

## 2 Results

This study investigated whether an anaerobic methane-oxidizing enrichment culture was able to transfer electrons to a carbon cloth electrode poised to a potential of +400 mV vs. standard hydrogen electrode (SHE). Low-density current was produced with a maximum of 247 *μ*A/cm^2^ and a final stable current of ~6.5 *μ*A/cm^2^, which was attributed to several non-methanotrophic bacterial members of the community.

### 2.1 Electrons are transferred to the extracellular acceptor graphene oxide

Graphene oxide can act as electron acceptor. Due to its size the molecule cannot enter cells and therefore has to be reduced extracellularly [36]. Graphene oxide was used in batch incubations together with ^13^C-labeled methane to probe the EET potential of our enrichment culture, a method that was previously used by Ref. [37] to determine whether a culture can perform EET. Three different experiments were performed with two replicates per experiment: abiotic control without biomass but with graphene oxide, graphene oxide and biomass, and nitrate (0.3 mM) and biomass as positive control. The conversion of ^13^C-labeled methane was measured by detecting ^13^CO_2_. While methane was the sole electron donor, the production of ^12^CO_2_ can still occur e.g. through the oxidation of storage compounds or dead organic matter. Therefore, the ^13^CO_2_/Total CO_2_ ratio was determined in order to reliably measure methane oxidation. For the abiotic control, no ^13^CO_2_ was produced from ^13^C-labeled methane. For the experiment with graphene oxide and biomass, the ^13^CO_2_/Total CO_2_ ratio increased from 1.2 to 2.6 and for the positive control from 1.2 to 3.7, showing the oxidation of labeled methane under both conditions. This does not exclude the possibility of acetate production from methane and its subsequent oxidation, or reduction of graphene oxide through the oxidation of decaying biomass from other microbial community members. To confirm the reduction of graphene oxide, samples were submitted to Raman spectroscopy (Fig. 1). Its reduction was indicated by the characteristic 2D and D+D’ peaks [38]. These peaks were detected in samples incubated with methane and graphene oxide, but not in the abiotic control.

**Fig. 1:**
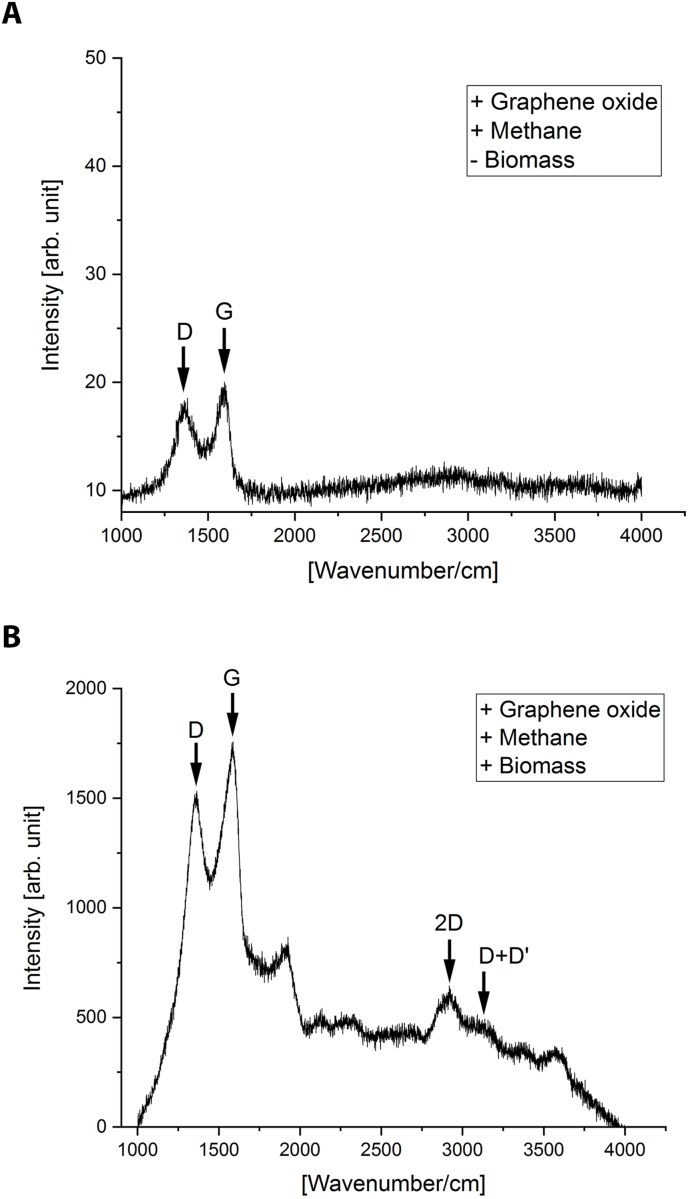
Raman spectroscopy of batch incubation with methane as electron donor and graphene oxide as electron acceptor. Abiotic control without biomass (**A**) and sample with biomass (**B**). The presence of 2D and D+D′ peaks (arrows) in the sample as compared to the control indicated reduction of graphene oxide with methane or a methane-derived substrate.

### 2.2 Bioelectrochemical analyses

#### 2.2.1 Setup of a two-chamber three-electrode system

The anaerobic methane-oxidizing community was maintained in a 10 L sequencing batch reactor producing granular biomass as previously described [39]. In order to apply the culture to a bioelectrochemical system (BES), the glass vessels, optimal culture volumes, the membrane and all electrode materials were tested and optimized. A classical H-cell in which the two chambers were connected via a Nafion cation exchange membrane was found to be most suitable (suppl. fig. 1). An AgCl reference electrode was used, with a carbon cloth working electrode and a stainless-steel counter electrode. These electrodes were connected via a platinum wire. Formation of a biofilm on the working electrode was promoted by manually breaking granular biomass to produce planktonic cells in a 15 ml glass homogenizer prior to inoculation of the system. In order to maintain anoxic conditions and to supply ample methane, the anode chamber was sparged with a mixture of CH_4_/CO_2_ in a 95:5 vol/vol ratio or with Ar/CO_2_ 95:5 vol/vol ratio when acetate was tested as an alternative substrate.

#### 2.2.2 Current was produced and cyclic voltammetry scans confirmed the presence of redox centers in the electrode biofilm

**Fig. 2:**
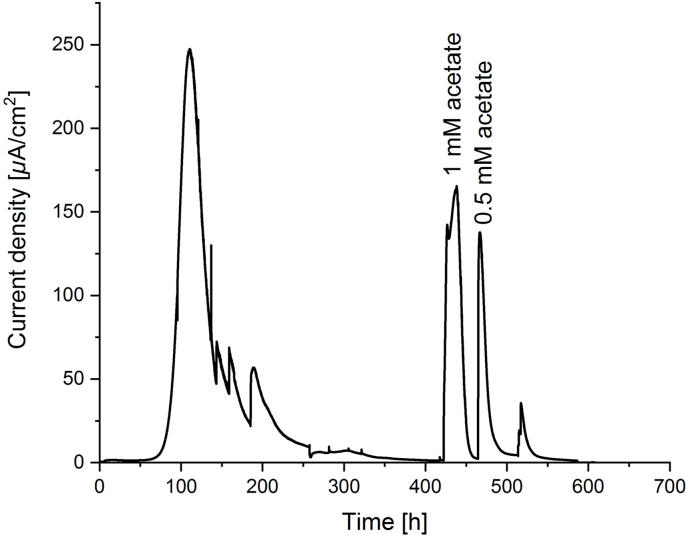
Current generation from an anaerobic methane-oxidizing community in a bioelectrochemical system with an applied potential of +400 mV vs. SHE. The anode chamber was sparged with Ar/CO_2_ and without any added substrates current density was between 5 and 8 *μ*A/cm^2^. Upon addition of acetate, current densities reached up to 165 *μ*A/cm^2^.

**Fig. 3:**
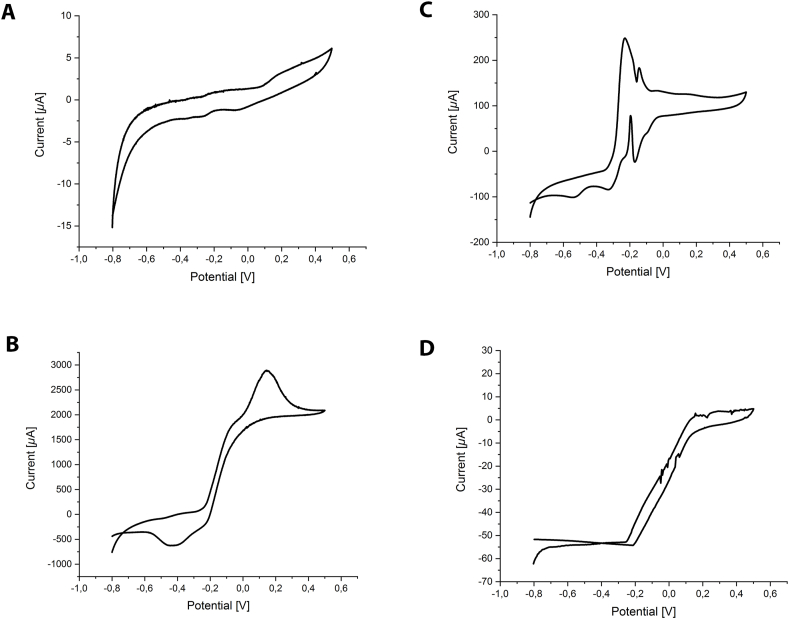
Cyclic voltammograms recorded after inoculation (**A**), during turnover conditions (**B**), during non-turnover conditions (**C**) and with cell-free spent medium (**D**). High current was observed during turnover conditions in accordance with substrate being oxidized and electrons transferred to the anode. In non-turnover conditions potential redox centers have been identified at −160 mV and −230 mV in the anodic and cathodic direction as well as at −330 mV and −530 mV in the cathodic direction. No peaks were observed with the spent medium, indicating that no soluble mediators were present in the spent media. All potentials are reported vs. SHE.

In the previously described BES, a potential of +400 mV vs. standard hydrogen electrode (SHE) was applied as this is close to the standard redox potential of the nitrate/nitrite couple (+420 mV) [40]. A visible biofilm developed on the working electrode surface. The production of low-density current was observed in four biological replicates. Generally, the same pattern could be observed (Fig. 2), which included no or very little current production at the start of a batch. This was expected as time is needed for the colonization of the working electrode and the formation of a biofilm. A sharp current peak was observed at 68–192 h with an increase from 6 to 247 *μ*A/cm^2^. Current stabilized at low densities of 5–8 *μ*A/cm^2^. Interestingly, the same pattern was observed in BESs sparged with CH_4_/CO_2_ or Ar/CO_2_. Hence, the current could be generated independently of methane or other externally added substrates. No current was produced in abiotic controls performed prior to inoculation and in controls that were sterilized by autoclaving after the end of a batch. The presence of redox centers was investigated by cyclic voltammetry (CV) scans (Fig. 3). Cyclic voltammetry scans were recorded at the beginning of a batch, at the highest current density, and after the liquid phase had been exchanged with fresh medium. Additionally, a CV scan was recorded using the cell-free spent medium to investigate the presence of soluble mediators. Several peaks were consistently present in all replicates. At the beginning of an experiment, no peaks were observed, which is in accordance with no or very low current density and the absence of a biofilm on the anode (Fig. 3A). At the highest current density (Fig. 3B, after appr. 100 h) high currents were observed, indicating substrate turnover. While scanning in the anodic direction, a peak at 140 mV vs. SHE was observed when maximal electron transfer was reached. Upon scanning after medium exchange, and hence in non-turnover conditions, the current was considerably lower and peaks potentially representing redox centers in the biofilm could be revealed (Fig. 3C). In anodic direction, peaks were detected at −140 mV and −230 mV. In the cathodic direction a peak was detected at −160 mV and an additional shoulder at −230 mV. These corresponded well to the two anodic peaks and might therefore represent the same redox centers that can reversibly be oxidized and reduced. Two additional shoulders at −330 mV and −530 mV in the cathodic direction with no anodic counterparts could represent additional redox centers within the biofilm. No peaks were observed in the scans of cell-free spent medium of a batch producing a stable current, which indicates that no soluble mediators where involved in EET (Fig. 3D).

### 2.3 The archaeal and bacterial communities change in response to the applied potential

**Fig. 4:**
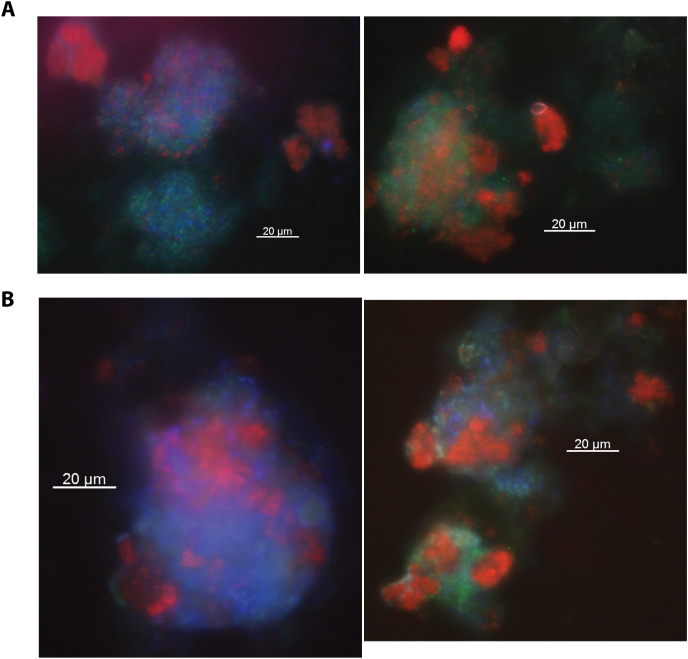
FISH micrographs of reactor biomass (**A**) and the anodic biofilm community (**B**). Cy3 – red: ‘*Ca*. Methanoperedens sp.’. Fluos –green: ‘*Ca*. M. oxyfera’. Cy5 – blue: general bacteria. It was shown that ‘*Ca*. Methanoperedens sp.’, ‘*Ca*. M. oxyfera’ and other bacteria were part of the reactor biomass as well as the anode biofilm community. (For interpretation of the references to color in this figure legend, the reader is referred to the Web version of this article.)

It was confirmed that current was produced by the biofilm community that grew on the working electrode. Hence, we investigated which organisms were potentially conducting EET. The community composition of the biofilm was investigated by fluorescence *in situ* hybridization (FISH) and metagenome sequencing. Target-specific FISH probes confirmed the presence of the anaerobic methanotrophs ‘*Ca*. Methanoperedens sp.’ and ‘*Ca*. M. oxyfera’ in the biofilm (Fig. 4). The presence of bacteria was confirmed using a general bacterial FISH probe. To gain deeper insights, samples were collected from both the biomass used for inoculation and the electrode biofilm. Their DNA was extracted and sequenced.

Trimmed reads of both the inoculum and the biofilm were co-assembled. The metagenomes were obtained from one bioreactor and one biofilm of a BES. The metagenome was binned using a consensus binning strategy, which resulted in the recovery of 41 metagenome assembled genomes (MAGs), most being more than 70% complete according to CheckM assessment. About 10–15% of the reads could not be aligned to any MAG. Taxonomic classification was performed using the GTDB-Tk taxonomic classifier (suppl. Table 1) [41]. The metagenomic reads as well as the recovered MAGs have been deposited in the NCBI database as BioProject PRJNA573876. ‘*Ca*. Methanoperedens sp.’ was the only archaeon detected in the dataset. Furthermore, 40 species of bacteria were identified. Proteobacteria and Bacteroidetes were the dominant phyla, e.g. 41% of the MAGs were identified as Proteobacteria and 22% of the MAGs were identified as Bacteroidetes. Changes in relative abundance, based on the mapped reads, were compared between inoculum and biofilm samples. Members of the biofilm community that had an abundance of at least 2% of the mapped reads and a fold change of at least 22-fold are highlighted in Table 1. Some bacterial species showed significant increases in the electrode biofilm compared to the inoculum. These included Betaproteobacteria classified as *Zoogloea* sp. and *Dechloromonas* sp. *Zoogloea* sp. increased from 0.01% relative abundance in the inoculum to 7.2% in the electrode biofilm. *Dechloromonas* sp. was likewise 0.01% of the original community and increased to 2%. A species that was classified as *Leptonema illini* (Spirochaetes) also significantly increased from 0.13% to 2.9%. Two bacteria could be classified only to the taxonomic level of phylum and belonged to the Bacteroidetes (Bacteroidetes_1 and Bacteroidetes_2). Bacteroidetes_1 increased from 0.08% to 3.1%. Bacteroidetes_2 was not detected in the inoculum and its abundance thus below the detection limit. This MAG made up 3% of the final electrode biofilm community.

**Table 1:** Relative abundance (% mapped reads) of the microbial community members in the inoculum and in the biofilm. The microorganisms are shown that increased a lot from inoculum to biofilm together with the methanotrophs.

Taxonomic classification	% mapped reads in control	% mapped reads in electrode biofilm	Fold change
*Zoogloea* sp.	0.01	7.2	720
*Dechloromonas* sp.	0.01	2	200
Bacteroidetes_1	0.08	3.1	39
*Leptonema illini*	0.13	2.9	22
Bacteroidetes_2	N/A	3	N/A
‘Ca. M. oxyfera’	11	10	0.9
‘Ca. Methanoperedens’	16	7	0.5

The number of reads assigned to the ‘*Ca*. Methanoperedens sp.’ MAG initially amounted to 16% of all reads and to 11% for ‘*Ca*. M. oxyfera’, respectively. In the anode biofilm ‘*Ca*. Methanoperedens sp.’ had a lower relative abundance with approximately 7% (Table 1). The relative abundance of ‘*Ca*. M. oxyfera’ was the same for both the inoculum and the biofilm. ‘*Ca*. Methanoperedens sp.’ grows slowly with a doubling time of 30–60 days [42]. The conditions in the BES did not enrich for either ‘*Ca*. Methanoperedens sp.’ and ‘*Ca*. M. oxyfera’, but both species still constituted a significant part of the biofilm community.

Initially, methane was the sole added substrate. However, the bacteria whose relative abundance increased significantly in the electrode biofilm compared to the inoculum are not known to directly use methane as substrate. Hence, the question arose if another substrate was being internally produced in the BES. In order to test whether the BES was running independently of methane, the methane supply was exchanged for an Ar/CO_2_ mixture (95:5 vol/vol). As a result, the registered current was stable. This was confirmed in a batch running with only Ar/CO_2_ and no methane. The enrichment culture used in this study originates from an anoxic sediment sample [43]. Generally, in these environments, various organic compounds are present with acetate as one of the most abundant short chain fatty acids [[44], [45], [46]]. In metal-reducing conditions, acetate has been established as substrate used by different species among which are the model organism *Geobacter* spp [47]. and also *Shewanella algae* [48]. In order to test whether current might be produced from another carbon source such as acetate, we added low concentrations (0.5 mM or 1 mM) to a running batch. A sharp increase in current production was observed (Fig. 2). Current densities of up to 165 *μ*A/cm^2^ could be reached by the addition of 1 mM acetate. To exclude that this was an artefact, the batch was autoclaved after the end of the experiment, re-connected and subsequently, the same amount of acetate was added. No current was observed, which supports current generation by live cells. The coulombic efficiency was calculated to investigate whether the current produced was in accordance with the amount of substrate provided. Adding 1 mM of acetate in a 300 ml volume theoretically provides 0.0024 mol of electrons. The measured current equaled transfer of 0.0022 mol of electrons and was hence in good agreement with the amount of substrate provided. Acetate is therefore possibly one of the most important substrates used by the biofilm community. Acetate could have been derived from decaying biomass or produced by ‘*Ca*. Methanoperedens’ archaea, which were recently shown to generate acetate from internal storage compounds [10]. Acetate can be generated from CO_2_ by acetogens using the Wood-Ljungdahl pathway (WLP), or it can act as intermediate or end-product of the fermentation of, for example, sugars and alcohols by acetic acid bacteria. Analysis of the annotated MAGs recovered from the metagenome showed that only the ‘*Ca*. Methanoperedens’ genome contained a full set of genes comprising the WLP, including the acetyl-CoA synthase/CO dehydrogenase complex (ACS/CODH). No acetic acid bacteria were detected in the metagenome. Acetate consumption can proceed through a reverse WLP-like pathway, which requires the ACS/CODH complex, or through activation of acetate to acetyl-CoA by either acetyl-CoA synthetase (ACSS) or through the combined action of acetate kinase and phosphate acetyltransferase. Only 10 MAGs lack all pathways for acetate activation: “*Ca*. Dadabacteria bacterium”, *Paludibacter* sp., “*Ca*. Brocadia sp.”, *Anerolinae* bacterium, *Melioribacteraceae* bacterium, *Sphingopyxis terrae*, Flavobacteriales bacterium, Bacteroidales bacterium, Bacteroidetes bacterium, and “*Ca*. Methylomicabilis oxyfera”, indicating that the trait of acetate utilization is widespread in the microbial community investigated in this study.

### 2.4 Bacterial MAGs contain genes involved in acetate oxidation and EET

The most abundant MAGs were annotated and checked for genes potentially involved in acetate metabolism and EET. All bacterial MAGs contained genes involved in the TCA cycle that provides a possible route for acetate oxidation. All MAGs except for Bacteroidetes_2 also contained genes encoding enzymes that convert acetate into acetyl-CoA, which is an intermediate needed to channel acetate into the TCA cycle.

**Fig. 5:**
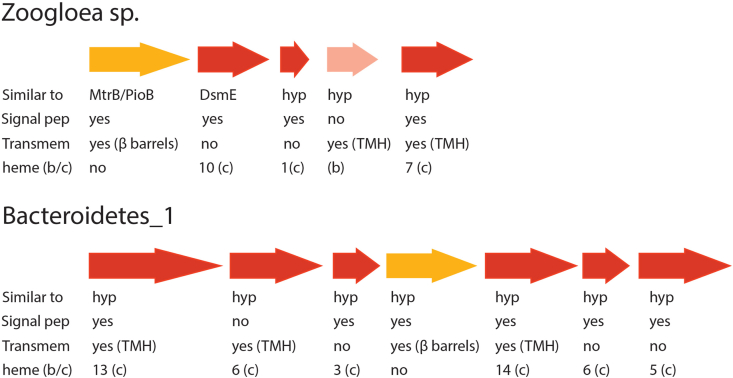
*Zoogloea* sp. and Bacteroidetes_1 gene clusters involved in EET. A cluster with five genes containing MtrB/PioB and DsmE homologues was identified in *Zoogloea* sp. A similar cluster was detected in Bacteroidetes_1. However, sequences matched only hypothetical entries in the NCBI database. Hyp: hypothetical protein, TMH: transmembrane helices, dark red arrow: *c*-type cytochrome, light red arrow: *b*-type cytochrome, orange arrow: porin. (For interpretation of the references to color in this figure legend, the reader is referred to the Web version of this article.)

EET in bacteria has been shown to proceed via multiheme cytochromes and some systems such as the metal-reducing pathway (Mtr) of *Shewanella oneidensis*, the porin-cytochrome pathway (Pcc) of *Geobacter sulfurreducens*, the metal-oxidizing pathway (Mto) of *Sideroxydans lithotrophicus* and the phototrophic iron oxidation (Pio) pathway of *Rhodopseudomonas palustris* [24]. While these pathways have originally been described to function either in an oxidizing or reducing direction, they can be bidirectional [49]. Mostly, these systems consist of a protein residing in the cytoplasmic membrane that is able to oxidize or reduce quinones and transfer electrons to proteins functioning as electron shuttles in the periplasm. Electron transfer through the outer membrane is facilitated by a porin that makes the membrane permeable for cytochromes that can in turn release electrons through direct contact to extracellular compounds [50]. In the 95.5% complete *Zoogloea* sp. MAG a gene cluster was identified that consisted of five genes that can be linked to EET (Fig. 5; suppl. tab. 2). The first gene in the cluster had 73% amino acid sequence identity to MtrB/PioB, which is the porin protein that facilitates electron transport across the outer membrane. Furthermore, it was identified as transmembrane *β* barrel protein with BOCTOPUS2 [51]. The second is a decaheme cytochrome that is highly similar to DsmE, which is involved in extracellular DMSO reduction in *Shewanella oneidensis* MR-1 [52]. No characterized homologues could be identified for the other three genes. They are however annotated as one *b*-type cytochrome and two *c*-type cytochromes, one of them a multiheme with seven CXXCH motifs. Additionally, according to analyses using SignalP [53] and TMHMM [54], signal peptides and transmembrane helices were present. Thus, *Zoogloea* sp. clearly has genomic potential for EET via proteins that are similar to those described in other gram-negative bacteria. This is in accordance with its enrichment on an anode as was found in our study.

In the *Dechloromonas* sp. MAG, which was 97.5% complete, there was less evidence for genes involved in EET as compared to the *Zoogloea* sp. MAG. Two multiheme cytochromes were identified, one containing four and one containing eight heme binding sites. Additionally, multiple proteins containing one or two heme cofactors were identified. Potential porins were also present but none of them showed high similarity to porins previously described to participate in EET. Conductive type IV pili can be used in another EET mechanism that has been well described for *Geobacter* spp [[55], [56], [57]]. Compared to non-conductive pili, the PilA protein is truncated to allow tight packing of aromatic amino acids generating a conductive biomaterial through π-π-stacking. In the *Dechloromonas* sp. MAG the machinery for type IV pilus assembly is encoded. Aromatic amino acids are mostly conserved in the protein most similar to *Geobacter* PilA (BALAKLNP_00253; suppl. tab. 2). However, it is considerably longer, therefore conductivity is questionable and needs to be proven experimentally.

The Bacteriodetes_1 MAG was 97.3% complete and contained a cluster that comprised seven genes that matched hypothetical proteins in the NCBI non-redundant protein sequences database (Fig. 5; suppl. tab. 2). All highly similar hits belonged either to the Bacteroidetes in accordance with taxonomic classification or to Ignavibacteriae, a phylum within the Bacteroidetes/Chlorobi group. Six genes encoded multiheme cytochrome proteins. Five of these proteins contained a signal peptide according to SignalP [53] and in three of these proteins, transmembrane helices were identified by TMHMM [54]. The only non-cytochrome protein was identified as a transmembrane *β* barrel protein with BOCTOPUS2 [51], making it a potential porin. This gene cluster likely is a previously uncharacterized gene cluster containing genes involved in EET. This makes it an interesting target for future studies.

*Leptonema illini* has so far not been linked to EET and genomic evidence for EET is scarce. In the 98.8% complete MAG only one gene encoding a porin, which was not similar to MtrB-PioB, could be identified and an incomplete set of pili assembly genes was found. One octaheme and one pentaheme with no well-characterized homologues could be identified at different genomic locations. Hence, it is questionable if *Leptonema illini* indeed participated in EET in the electrode biofilm. The Bacteroidetes_2 MAG with a completeness of 95.2% contained two multiheme cytochrome encoding genes, no porin homologue and also no genes involved in the formation of pili. Hence, similar to *Leptonema illini*, it is not likely that this organism has the potential to participate in EET.

## 3 Discussion

In this study, an anaerobic methane-oxidizing community was investigated with regard to its potential to perform EET. We observed that the culture could use ^13^C-labeled methane as substrate with concomitant reduction of graphene oxide. Since graphene oxide cannot enter cells but is reduced extracellularly, this indicated that electrons from methane, or a methane-derived intermediate, or decaying cell biomass are transferred to graphene oxide via an EET mechanism. Probably, the mechanism of EET was direct as there was no indication of the presence of soluble mediators at the end of the experiment in the cell-free spent medium.

To further investigate this, we used a BES with an applied potential of +400 mV vs. SHE. It was found that the enrichment culture could transfer electrons to the anode, which was measured as a positive current. Methane was oxidized with graphene oxide as electron acceptor, demonstrating the potential of the community to perform EET with methane as electron donor. The electrode with a potential of +400 mV did not enrich for the methanotrophs, *‘Ca.* Methanoperedens’ and ‘*Ca.* oxyfera’, in the biofilm. In contrast, several bacteria including *Zoogloea* sp., *Dechloromonas* sp. and a member of the Bacteroidetes, enriched at the biofilm and may have been largely responsible for the produced current. Alternative electron donors may have led to the enrichment of these bacteria.

These alternative carbon sources may have been derived from the biomass during the homogenization process, from decaying biomass in the anolyte, or from the conversion of methane into products. Interestingly, it has been speculated that ‘*Ca*. Methanoperedens nitroreducens’ is able to produce acetate via ACS/CODH and ACD [58]. This metabolic route is directly linked with reverse methanogenesis. Recently, evidence was provided for acetogenesis performed by ANME-2a, which are phylogenetically closely related to ANME-2d [59]. Additionally, it has been found that ‘*Ca*. Methanoperedens nitroreducens’ could produce acetate from methane-derived storage compounds such as polyhydroxyalkanoate and glycogen [86]. [86] demonstrated that in a nitrate-limited enrichment culture acetate concentrations reached 1.6 mM. We explored acetate as one of these possible carbon sources as this is one of the most abundant carbon sources in anoxic sediments where the enrichment culture originated from. Upon addition of 1 mM acetate to the anolyte, a high current density of 165 *μ*A/cm^2^ was reached which corresponded with the electron equivalents of 1 mM acetate. Hence, electrons were probably not traveling via methane oxidation by ‘*Ca.* Methanoperedens’ with concomitant transfer of electrons to the anode but possibly via an alternative carbon source such as acetate as an intermediate. If acetate is indeed the intermediate fueling EET in this community, ‘*Ca*. Methanoperedens’ is likely the only organism in the enrichment that can produce it, due to the lack of a complete WLP in the other MAGs. In contrast, only 10 out of 41 MAGs lack the genes required for acetate activation to acetyl-CoA indicating that the trait of acetate utilization is widespread. Coincidentally, different bacterial species capable of using acetate have been shown to increase in relative abundance in the biofilm. In addition, genes involved in EET have been identified in some highly enriched bacteria: *Zoogloea* sp., *Dechloromonas* sp. and one member of the Bacteroidetes. Interestingly, the Bacteroidetes MAG lacks both acetyl-CoA synthetase and the acetate kinase/phosphate acetyltransferase genes and appears to be incapable of acetate degradation, pointing to another yet unidentified substrate driving part of the current production.

Similar results have been obtained in two studies: in a microbial fuel cell fed with methane as sole electron donor and inoculated with a mixed community [60] and in a fuel cell using a synthetic consortium containing *Methanosarcina acetivorans* engineered to oxidize methane and produce acetate [61]. In the engineered community, acetate was taken up and used for current production by *G. sulfurreducens*. In the mixed community, weak electrogenic capacities were demonstrated, and ANME-2d archaea as well as *Geobacter* spp. and *Ignavibacterium* spp. increased in abundance. *Geobacter* is a model organism for EET (for a recent review please see Ref. [62]). For *Ignavibacterium,* participation in EET is also established, members of the genus *Ignavibacterium* have previously been found to be enriched under acetate-oxidizing and iron-reducing conditions [63]. Hence, links between methane-consuming/oxidizing microorganisms and electroactive bacteria have been observed.

Since it has been speculated that ANME-2d archaea are capable of extracellular electron transfer [[17], [18], [19], [20]], the question arises why electroactive bacteria were enriched that are not capable of using methane, while the BESs were fed with methane. Both methane and alternative carbon sources such as acetate are thermodynamically feasible electron donors for an electrode with a poised potential of +400 mV vs SHE. The redox potential of the methane/CO_2_ couple under standard conditions is −240 mV, which means that electron transfer to an electrode poised to +400 mV is highly exergonic with ΔG^0^’ = −494 kJ/mol. We take acetate as an example of other organic carbon sources: the acetate/CO_2_ couple has an even lower redox potential of −290 mV, here ΔG^0^’ is even higher at −533 kJ/mol. Hence, both processes are thermodynamically feasible, with acetate oxidation being slightly more favorable. Apart from thermodynamics, factors such as substrate availability, substrate affinity and doubling time have to be taken into account. Generally, with a K_M_ of approximately 10 mM for methane [64], substrate affinity is considered to be low in ANME archaea and due to the low solubility of methane in water, substrate availability is limited. Hence, due to these factors in combination with a long doubling time, ANME archaea can easily be outcompeted given the availability of substrates other than methane.

In our study, the ecological niche of substrate oxidation using an electrode as terminal electron acceptor was occupied by several bacterial species, including *Zoogloea* sp., *Dechloromonas* sp. and a member of the Bacteroidetes. *Zoogloea* spp. are commonly found in wastewater treatment plants. Several metabolic activities have been described, among which the oxidation of acetate with nitrate as electron acceptor [87]. This could explain how this organism can sustain itself in the nitrate-fed enrichment culture used for inoculation of the BES. Furthermore, *Zoogloea* has been linked to EET with AQDS [65] and has additionally been found in an electroactive biofilm [66]. The MAG of the *Zoogloea* species present in this study was investigated concerning genes potentially involved in EET. A putative porin similar to MtrB/PioB as well as multiple multiheme cytochromes and a potential DmsE homologue were found in this MAG. Hence, the *Zoogloea* species present in this enrichment likely has the potential to perform EET.

Another bacterium that was highly abundant in the anode biofilm was a *Dechloromonas* species. Similar to *Zoogloea* spp., *Dechloromonas* strain UWNR4 has been shown to be able to reduce nitrate, interestingly upon oxidation of Fe^2+^ with concomitant acetate consumption [67]. Adding to that, homologues of the Mtr pathway, which is well-studied in the EET model organism *Shewanella oneidensis*, have been identified in *Dechloromonas aromatica* RCB [49], providing evidence that *Dechloromonas* spp. are potentially capable of EET. In a recent study ‘*Ca*. Dechloromonas occulta’ was enriched from Lake Matano sediments [68]. Similar to *Zoogloea* and other *Dechloromonas* strains, denitrification genes are present in the genome. The authors noted that EET takes place with Mn(III) but not Mn(IV) as electron acceptor involving the previously described Mto proteins together with novel multiheme cytochromes encoded in the *occ* gene cluster. Interestingly, methane in culture headspaces promoted extracellular Mn(III) reduction as well as expression of genes encoding cytochromes and structural components such as S-layer proteins involved in manganese binding. The genomic potential of the *Dechloromonas* sp. MAG assembled in this study was investigated with respect to genes potentially participating in EET. Several cytochromes were identified, among which are two multiheme cytochromes. Additionally, porin-encoding genes and a *pilA* homologue were found. Those were, however, not highly similar to porin/PilA proteins involved in EET. Therefore, while *Dechloromonas* spp. are generally able to perform EET, it is not entirely clear whether the *Dechloromonas* species studied here has this capacity.

Another highly abundant bacterium, here called Bacteriodetes_1, was more closely investigated. Due to the lack of accurate classification it is speculative to investigate EET in closely related bacteria. Members of the Bacteroidetes have, however, been linked to EET [[69], [70], [71]]. Furthermore, a gene cluster encoding genes potentially involved in EET could be identified in the Bacteroidetes_1 MAG. This cluster consisted of seven genes, six of which were classified as multiheme cytochromes. Predicted transmembrane helices and signal peptides indicated integration into the membrane and a possible role in externalization of electrons. The whole cluster is an interesting target for future studies.

Altogether, the enrichment culture used in this study could produce current at low densities. Biofilms of *G. sulfurreducens* produce the highest current density of all organisms tested in pure culture, comparable to current densities produced from mixed biofilms. Densities as high as 390–456 *μ*A/cm^2^ have been measured [72,73]. In this study current densities as high as 247 *μ*A/cm^2^ have been observed during peaks. Stable current, however, was much lower at around 5–8 *μ*A/cm^2^. It remains to be further investigated how substrate availability influences current production in our system. Potentially, in natural environments with a constant supply of methane, nitrate and manganese, stable co-cultures of acetate-producing ‘*Ca*. Methanoperedens spp.’ and metal-reducing bacteria exist, as was potentially observed in Lake Matano sediments [68]. More studies are needed to further disentangle the metabolic relationships between methane-oxidizing archaea and metal-reducing bacteria.

## 4 Materials and methods

### 4.1 Reduction of graphene oxide as proxy of EET

Batch assays were performed using graphene oxide as extracellular electron acceptor to probe for EET. The enrichment culture used for batch assays was maintained in a sequencing batch reactor as already described [39]. Reactor content was collected anoxically using an anoxic chamber (N_2_/H_2_ 95:5 vol/vol), and 50 ml of culture was transferred to a 120 ml serum bottle per experimental condition. Three different conditions were used with two replicates per experiment. For the abiotic control, biomass was removed with centrifugation and subsequent passage through a 0.2 *μ*m filter. For the second condition, graphene oxide was added as electron acceptor at a final concentration of 200 mg L^−1^ together with the biomass. As positive control, 0.3 mM sodium nitrate was added in addition to graphene oxide. The gas phase was exchanged with Ar/CO_2_ 95:5 vol/vol and 14 ml ^13^C-labeled methane was added to all bottles, corresponding to 20% methane in the headspace. Bottles were incubated at 28 °C while shaking. Gas chromatography – mass spectrometry measurements of ^13^CO_2_ (Agilent 6890 and 5975C inert MSD, USA) were done as described previously [43]. To confirm the reduction of graphene oxide, samples were centrifuged and subjected to dehydration with absolute ethanol. Samples were maintained in a desiccator until Raman spectroscopy analysis. Raman spectroscopy (StellarNet Inc) was performed with the following settings: Laser 473 nm, acquisition time 20 s, accumulation 5 and objective 50X.

### 4.2 Bioelectrochemical analyses

#### 4.2.1 Activation of Nafion cation exchange membranes

The two chambers of the BES were connected via a 0.002 inch Nafion cation exchange membrane (Sigma Aldrich, Zwijndrecht, The Netherlands). Nafion membranes were pre-treated by incubating at 60 °C in MilliQ water for 60 min, in 3% H_2_O_2_ for 60 min, in MilliQ water for 30 min, in 50 mM sulfuric acid for 60 min and in MilliQ water for 30 min. Activated membranes were stored in MilliQ water at 4 °C.

#### 4.2.2 Cultivation in the bioelectrochemical system

In order to measure EET in the form of positive current, a BES was used. For inoculation, 300 ml of enrichment culture was anoxically harvested. In an anoxic chamber with a N_2_/H_2_ 95:5 atm the biomass was homogenized using a 15 ml glass homogenizer until no more granules were macroscopically visible. The cell suspension was filled into custom-made glassware (Adams and Chittenden Scientific Glass, Berkeley, USA). The BES was assembled using a carbon cloth (Fuel Cell Earth, Woburn, USA) working electrode connected via platinum wire (Goodfellow, Huntingdon, UK), an Ag/AgCl reference electrode (ProSense, Oosterhout, NL) and a stir bar. The cathode chamber was filled with 150 mM potassium phosphate buffer pH 7.5 and equipped with a stainless steel mesh (Goodfellow, Huntingdon, UK) and platinum wire (Goodfellow, Huntingdon, UK) counter electrode. The anode chamber was maintained at 30 °C by connecting the glass jacket to a water bath and continuous sparging with either CH_4_/CO_2_ 95:5 or Ar/CO_2_ 95:5 via the lowest port (suppl. fig. 1) at a flow rate of 12 ml/min. For gas distribution the anode chamber was stirred at 100 rpm. Acetate was added to final concentrations of 0.5 and 1 mM with no methane present. In order to calculate the number of electrons transferred upon addition of 1 mM acetate, the integral of the measured peak was calculated, giving an area of 212 C. The number of transferred electrons was calculated knowing that one electron equals 1.6e-19 C and one mol of electrons equals 6.02e23 electrons. No nitrate was added and throughout the experiment nitrate concentrations remained under the detection limit of colorimetric test strips with a lower detection limit of 2 mg L^−1^ (MQuant test stripes, Merck, Darmstadt, Germany). All three electrodes were connected to a MultiEmStat3 potentiostat (PalmSens, Houten, the Netherlands) and a potential of +400 mV vs. SHE was applied. Current generation was measured for 21 days on average and monitored via the MultiTrace software (PalmSens, Houten, the Netherlands) in chronoamperometric detection mode with measurements taken every 60 s.

#### 4.2.3 Cyclic voltammograms

Cyclic voltammetry was performed to investigate the electrochemical properties of the anode biofilm and the cell-free spent medium of a batch producing stable current. Voltammograms were recorded via a MultiEmStat3 potentiostat (PalmSens, Houten, the Netherlands) in cyclic voltammetry mode performing duplicate scans with scan rate 0.001 V s^−1^. The potential range was −800 to +500 mV vs. SHE and scanning was performed first in anodic and then in cathodic direction. During scans, sparging and stirring were stopped. For the cell-free spent medium the liquid phase of a batch producing stable current was removed inside an anoxic chamber with N_2_/H_2_ 95:5 vol/vol atmosphere without disturbing the biofilm. Planktonic cells were removed by centrifugation (10.000×*g*, 15 min, RT) and a CV scan was recorded as described above.

### 4.3 Fluorescence *in situ* hybridization

By using FISH, the microorganisms present in the sample were assigned to a phylogenetic group by fluorescent labeling. For FISH analysis biomass was sampled from the working electrode at the end of a batch and fixated by incubating in paraformaldehyde fixative for 30 min at room temperature. All other steps were carried out as described in Ref. [9]. For ‘*Ca.* Methanoperedens’ a Cy3-labeled probe was used with the sequence ACTGDTAGGCTTGGGACC, for ‘*Ca.* M. oxyfera’ a Fluos-labeled probe was used with the sequence 5′-GACCAAAGGGGGCGAGCG-3′ and for general bacteria a Cy5-labeled probe was used with the sequence 5′-GCTGCCTCCCGTAGGAGT-3’.

### 4.4 Metagenome sequencing

In order to create an overview of the different bacterial and archaeal species present in our samples, total DNA was extracted, sheared, sequenced and assembled back into MAGs. Biomass was harvested by centrifugation (5 min, 10.000×*g*) from the material used for inoculation for the control and by washing of the working electrode and harvesting by centrifugation (5 min, 10.000×*g*) for the biofilm. DNA was extracted using the Power soil DNA extraction kit (Qiagen, Venlo, The Netherlands). DNA was quantified using the Qubit dsDNA HS Assay Kit (Thermo Fisher Scientific, Ochten, The Netherlands) and all DNA purification steps were performed using AMPure XP beads (Beckman Coulter, Brea, USA). To shear genomic DNA and add adapters in the same step (“tag-mentation”), the Illumina Nextera XT library prep kit (Illumina, San Diego, USA) was used. Afterwards, quality and size distribution were analyzed using the 2100 Bioanalyzer (Agilent Technologies). The library was normalized to 4 nM and sequenced with an Illumina MiSeq instrument using the manufacturer's 300 paired-end sequencing protocol. For the control 10.4 million reads were obtained and for the biofilm sample 10.7 million. Quality-trimming, sequencing adapter removal and contaminant filtering of Illumina paired-end sequencing reads was performed using BBTools BBDuk version 38.16. Processed reads for all samples were co-assembled *de novo* using metaSPAdes v3.12.0 [74] at default settings. MetaSPAdes iteratively assembled the metagenome using k-mers of length 21, 33, 55, 77, 99 and 127. Reads were mapped back to the assembled metagenome for each sample separately using Burrows-Wheeler Aligner 0.7.17 (BWA) [75], employing the “mem” algorithm. The sequence mapping files were processed using SAMtools 1.7 [76]. Metagenome binning was performed for contigs greater than 1500 bp. To optimize binning results, four different binning algorithms were used: BinSanity v0.2.6.3 [77], CONCOCT 0.4.1 [1], MaxBin 2.0 2.2.4 [78] and MetaBAT 2 2.12.1 [79]. The four bin sets were supplied to DAS Tool 1.1.1 [80] for consensus binning to obtain the final MAGs. The quality of the MAGs was assessed through a single-copy marker gene analysis using CheckM v1.0.11 and taxonomy was assessed using the classify workflow (classify_wf) of GTDB-Tk v0.3.0 [81,82]. For community analysis MAG coverage data was normalized by using sequencing depth per sequencing dataset and MAG averaged contig read coverage data generated by BWA.

### 4.5 Analysis of key genes involved in extracellular electron transfer

MAGs obtained from metagenome sequencing were annotated and further analyzed in order to find genes encoding proteins potentially involved in EET. Initial annotation of the MAGs of interest was performed using Prokka 1.13.7 [83]. The protein FASTA files produced by Prokka were subjected to an additional round of annotation by the EggNOG 5 server [84] using eggnog-mapper 2 [85]. Additionally, the protein FASTA files were mined for the presence of putative multi-heme c-type cytochromes by searching for the heme c binding motif CXXCH using a regular expression (C[A-Z][A-Z]CH) with python 2.7.12. Protein sequences of interest were further investigated using SignalP 5.0 [53], to predict the presence of signal peptides as well as the likely localization of the protein, and TMHMM 2.0 [54], to predict putative transmembrane helices.

## Author contributions

SB and DRS performed experiments. SB, TB, JF, HO and MitZ performed data analysis. SB, JR, MJ and CUW planned the experiments. SB wrote the paper with contributions from all coauthors.

## Declaration of competing interest

The authors declare that the research was conducted in the absence of any commercial or financial relationships that could be construed as a potential conflict of interest.

## Figures and Tables

**figs1:**
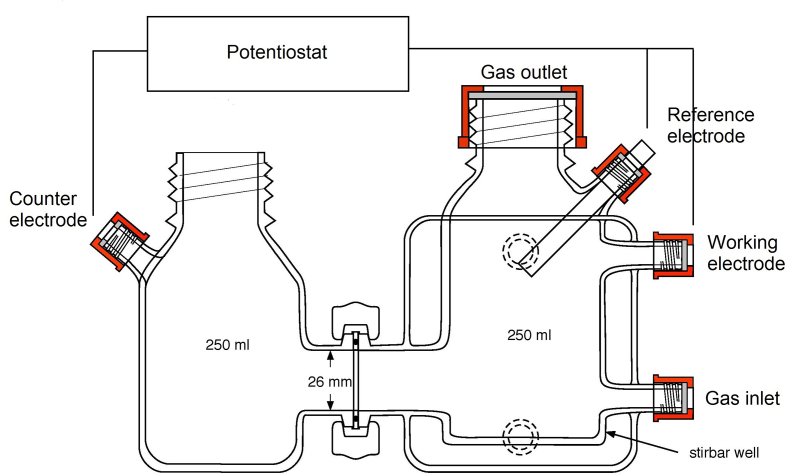


## Footnotes

Appendix A Supplementary data to this article can be found online at https://doi.org/10.1016/j.bioflm.2021.100054.
