# Supplementary material for: Current production by non-methanotrophic bacteria enriched from an anaerobic methane-oxidizing microbial community
Source: Biofilm. 2021 Jun 15;3:100054. doi: 10.1016/j.bioflm.2021.100054 (PMC8258643; doi:10.1016/j.bioflm.2021.100054)
Supplement: Multimedia component 2 [file mmc2.docx]

Supplementary table 2: Protein sequences derived from annotated MAGs potentially involved in extracellular electron transfer in *Zoogloea* sp., *Dechloromonas* sp. and Bacteroidetes_1

| **MAG / similar to** | **Protein sequence** |
| --- | --- |
| *Zoogloea* sp. / MtrB/PioB | >PMGACJAN_00642 MKSHSIFRPTLLAIALMAAFPTVSAWADDDVAELTSPNATDATLNLPYTDKVNPLYRQYNATTKEGISPNVDVDIVRRKDAEWFSVNARNLGLRTQEAGISYEKQGDWSISLDYNQIPRYSPYDVRTAVSGVGSTTIGQPNIANTAAAANNPNLYDVTLRTEREITTVAGSKYVMPGLKLGFSVKNEDKTGTRMSGVRGVTGTGATPANIYSAFLFAPEPINQNHKQMEATVEYTTEKYQLSAGFYGSFLGTKQNALAVVPGTNTALVATNLSPIALAPDNSVQQFYAAGAYNFSKDTRANLKVAWSEGRQDDNFLTGQPTLAGIGSSLNAKVQTTEVYSSITSRLTKDLKVLASWRYEDRQDKTPIRIFGTTTSNGVTTNYLNNPESHTANWGKLEANYRLGAGFNLTGGFDYSNKSSKEWERHDVSELTSRLAVSHSMGESLNGTLSFAHSERKGSEWNNGTTPPILPVYLADRNRDRIRGMLDVTATEALNIQFAYEAYLEDYNKSTYGLDKGQGQIFSVDSSYAISDVWKLNAWYTKQNGETRQYAQGAVCTTGNGSNCTVNTFRTGTLVQWDANLKQDSDQFGIGLNGRIARVDVGAQLLIYQDVNKQEVSKMPATTCTNATCSTTGTVAAGMGVLPDTKYTQNTFKLFGMYPVSKATRVRLDYIYDLRKMDDYTWTNWVYADGTRVYVKPEQTTQVIGLSLLHSF |
| *Zoogloea* sp. /  DsmE | >PMGACJAN_00643 MKFLRQLFASCLVLGALAGASVAPAADTPAGAKPAAKDLVLKGDAKCTSCHDESDEPKLLHIGKTKHGTVADGRTPSCTSCHGESDLHTNNPQKLKDRPATDRNFGKGSKLTAEEKSAACTTCHQGGKHMNWNTSAHANRDVGCTSCHQVHVEKDKVRDKRAQAEVCYTCHKQQRSELNRPSHHPIPEGKMSCSDCHNAHGSAGPKMLVKDTTNATCFTCHAEKRGPFVHNHQPVVEDCGNCHNPHGTTAEAMLKARQPFLCQQCHADASHPGNVPAIRANMPNAVSSNIGPGYAQARGCANCHTNIHGSNSPSNATSSGPFRFFR |
| *Zoogloea* sp. /  hypothetical proteins | >PMGACJAN_00644 MKKLKLHALVMSGLCAAGLLASSAALAFDADEAQALAKKEGCFKCHALDKKKEAKSLTDISKSLKGKSDAEAKLLHHLTVPEMVKFEDGKEEEHKILKTKDKAAIKNLTDWILSLAK |
| *Zoogloea* sp. /  hypothetical proteins | >PMGACJAN_00645  MTKRVFMLPLWIRLWHWSNALAIIVLAVTGMSLHFSDPALPLVEFSLAARIHNVAGVILVGLYAVFVIGNIVTGNWWQYVPKPPGIIQRCLRQMQYYGSGIFKGEPEPFPPTPETNFNALQAVTYWSIMYLVLPAVIVSGLIFLYPQFSPDTLFGLDGLLPIALVHYLGAAVIILFVVSHIYLGTMGPKVSSLFKMMFTGWYEH |
| *Zoogloea* sp. /  hypothetical proteins | >PMGACJAN_00646  MIGHFRKTLWRILVLLAVLAAAPALADDPPSPDELAKIRTGNAACMACHSEAGLKKPPKEGVDLKALRKYLVHADTYAASDHGQMACTKCHGDGYDNHPHAAKAREGLSECQDCHARKAMRIERQFDKSVHAENLSDTFTCATCHDAHTMALASKLRDPHKIVAQDNKICLDCHDSDLAFARIAPEKKKRPPIDDIHDWLPNTRLHWKAVRCIECHTPAEDKLSLSHEIQNKDKAEKKCATCHSANTSLNARLYRHLATEEQHKYGFINSVILGSSYVVGATRNPTLDFGLIILFAATVVGVLGHGLVRIITTRLRRSKKND |
| *Dechloromonas* sp. / PilA | >BALAKLNP_00253  MKRIQQGFTLIELMIVVAIIGILAAVALPAYQDYTVRAKVSEVVLAASGGRTTIAEAFQTLGHMPAVASAGLSSQNSKFVSAVDYTTTATNVGIITATAQGEPKITGSTIVMTGTADASGVVQWVCSGTIDPKYKPANCR |
| Bacteroidetes_1 / hypothetical proteins | >JNFKJAOJ_00295  MKLRSTLLAVALLAGLLSAQTKEECLACHSDNSLTMEKKGKTVSLFTDEKHLNASPHKKLSCTACHTNFDPNNLPHKENIAPVQCATCHAKDLPKHTFHKTVLDDGKDKSAMCKDCHGSHDVVSPKVAGAKFNKATIVEDCGQCHSDEKDHFLASAHGLAFSSKVPNAPSCLDCHSRLLPSSVKSDDAAQLKLQQEKMCMSCHGSAEMTQGVASSFIHAYEQSVHANALKNGNGKAANCVDCHSSHDMQKGSHPDSKVNKKNIPGTCAQCHEGVVKTYETSIHGKAFAGGNTVSPVCTDCHGEHKILGTKDPDSPVSKLHVAEEVCAPCHASVKMSEKFGLPTGKTNTFNDSFHGLAVKSGSKEAANCASCHGYHDILPSSDPASRVSKSNLAKTCGTCHPGAGENFSAGVVHVSTKADSEDDLIAFVSNIYVVLILVTIGGMAAHNILDFVRKSKRRLQHRRHGPGEEEVGHSLYVRMTLGERLQHGALALSFITLVVTGFMLRYPDAWWVVGIRSLSESVFEIRGVIHRVAAVVMVLASLYHLYYIFFVPRGKQLIRDLLPKVQDAYDAIGVARYNLGLSKEKPLLDRFSYIEKAEYWALIWGTIVMAVTGVILWFDVTFINLIGKQLWDVSRVVHYYEAWLATLAIIVWHFYFIIFNPDVYPMNLAWLKGTISEEEMADEHPLELRRIKEEQLRLEEEEKKKNADIN |
| Bacteroidetes_1 / hypothetical proteins | >JNFKJAOJ_00296 MKDLRSLLPTTFFNPVSYLGGAVSVASFILILFLFTVEMLGVPMQPYVGIITFVVLPGIMLLGIAVSLYGMRREKRRRLRTGSETAVFPTLDLNDPVQRTRFTFASILVFILLLGTAFGSYQVYHFTESVTFCGQICHQVMKPEFVAYSNSPHSRVTCADCHVGSGADWYVKSKLSGAYQIYSVIFKKYSQPIPTPVHSLRPAQGTCEQCHYPQHFHNDIQVNKTYYLKDEANTPWSVSLLMKISGGRSDSGPTSGIHWHVNRDHAVTYTAVDSQRQNIPVVRVRYPDASEDEFVTTDAY  DAAALAAGETRTMDCIDCHNRPTHIYRSPEETINAEMSHGAIAPTLPSVRLTVSEALTQEYADDEAAMKGIASYITDFYRLRHPALFASRRTEIDSAVSVAKRIYERNFFPYMRVSWKRYPNNIGHFNNLGCFRCHDNKHVSKKTGKVLSNDCNTCHTILSQGTSLASNISPKGLEFLHPVDIGDAWKTMNCIDCHTGQ |
| Bacteroidetes_1 / hypothetical proteins | >JNFKJAOJ_00297 MKKLLYLLFAVVIALPLAAQNKYVGVKGCACHNMPKQGKQVDVWKKSDHAKAFETLKSDKAAEIAKKNGIASAAEAKQCLECHSTGAGDPAEKTFAAADGVQCEACHGAASGYKAIHNKPENKEKAVAAGLLKADEKTCKPCHGANKMHDAKPFDYKKAWEQIKHPAPKG |
| Bacteroidetes_1 / hypothetical proteins | >JNFKJAOJ_00298 MERTLLAALVLLSMTADAQSINGRLTTSFYGFEGRDAALAKQTYLRAYENVYLNASSGDVSFNMNAMVSNDFGSDLATDPELRISSLLVKVRKIGGLADLSVGRQFIFAGAGYGLIDGAQTGLRFLDDRVSVTLYGGTNVDHTRDVRKQWIGSNGMFGGQVVFAPVENGSVGLSYMNKRRERAPYTAVRADSLFNPYIIVVNSTPLEEELASVDAEYEFGHAVMLQAKADYDVHHAELSRIQAFTRVHAMEGLSGTLEYIFREPRVAYNSIFSVFNTNSTQEIEGGLEYRHSPFFFLFARFADVQYVDDNSQRLSVGGTYEFLSAAYTQNFGYAGELNGVSLQAAYPLMDRVITPTCGFGYASYKHAKDDPSSTVVNLAAGAVYRPSKYISADVQLQWMQNPQFDSDMRAFVKFTYWFNDRLGWLE |
| Bacteroidetes_1 / hypothetical proteins | >JNFKJAOJ_00299  MKKNLTIAAAAAVLIGLVLFGRGTVVADDAAPADKTQLIKFSHAKHAEAGAECASCHKADLSDNSSDRMLPGHAECQSCHEQEVNENCSFCHTNPDDPQALPNPVRSLFFSHKQHVGMEGVKCETCHQGMDKADFAGHQNLPAMATCNTCHNDVKATNQCEACHTDLSNLRPASHNVANFKREHARVMSLRTFEAKCQSCHTEQSCAECHDGTNLTELAPGVKSGLLSPRHAAGDKAVALAGEAVHGMNYRFTHGIDAKGRAADCQTCHSSQQFCSDCHMNGSAALGGAMPTSHEAPGFTTIGVGSGGGSHATLAKRDIQRCMTCHDTEGGDPNCITCHADYDGIKGTNPRTHKSGFMKDNEHGEWHDDANANCFVCHTDANAKPSGRPGQGFCGYCHGVK |
| Bacteroidetes_1 / hypothetical proteins | >JNFKJAOJ_00300  MMNTTLRFGTLALVAALLLGGCAETKEDKVPTTPEVSVHGAGFADISSSNFHAKYIQGKNFDLSLCKTCHGPDFSGGTSGKSCNTCHSKPGGPENCTVCHGSVNAAPPFDLAGNVSPASRAVGAHQVHLLGGLVGAKVTCTECHVVPAALTAAGHIDNSAHAEVRFDPSSVFYKADAVFSGSGTTATCSNTYCHGNFPGGNGNVTMTWSDTNPDAAACGTCHGDVTKATFAEKAFPKSGHPAIGSMTCNQCHSRTVNSLAQIIDPSKHVNGVVD |
| Bacteroidetes_1 / hypothetical proteins | >JNFKJAOJ_00301 MKHLLTVLIALLFLALQACEGPEGPAGAQGPAGKDGANGQNAGFVYFEGFKDSLRCATCHTPDADTALFMAAKTLEYESAGHMEGTSWARGINSGDCASCHITEGYLESARGNFASQATKLYSHGSQPGCFTCHSPHKAGSFAVRKTDAIKVKSFVAGAADLQFNVGSSNTCVTCHRTRETSPMTNSVFVNGVSGNPDPTKTAATDSIEIKTSRFYPHYGVQGQILTGKGGFEFTGYTYPSSYHTNLAAAKNIQCSDCHMANPTVIVNGTPVAGGHTLKIGYSSISTDSANNMVNVAGCRTTGCHPSAFTANTVTATVFAAFKQSGQKALQDSMATLKSMLVSKGWLDPATDLAKLTGGKLVIKPAYKAGALFNYFFLEHEGSHGMHNMQYAQALLNASLAELRKP |
